# Functional Elucidation of *Nemopilema nomurai* and *Cyanea nozakii* Nematocyst Venoms’ Lytic Activity Using Mass Spectrometry and Zymography

**DOI:** 10.3390/toxins9020047

**Published:** 2017-01-26

**Authors:** Yang Yue, Huahua Yu, Rongfeng Li, Ronge Xing, Song Liu, Kecheng Li, Xueqin Wang, Xiaolin Chen, Pengcheng Li

**Affiliations:** 1 Key Laboratory of Experimental Marine Biology, Institute of Oceanology, Chinese Academy of Sciences, 7 Nanhai Road, Qingdao 266071, China; buqun08@163.com (Y.Y.); rongfengli@qdio.ac.cn (R.L.); xingronge@qdio.ac.cn (R.X.); sliu@qdio.ac.cn (S.L.); lkc@qdio.ac.cn (K.L.); xueqinwang@qdio.ac.cn (X.W.); chenxl@qdio.ac.cn (X.C.); 2 University of the Chinese Academy of Sciences, 19 Yuquan Road, Beijing 100039, China

**Keywords:** zymography, LC-MS/MS, enzymatic toxins, hemolysis, jellyfish metalloproteinase inhibitors, PLA_2_ inhibitors, *Nemopilema nomurai*, *Cyanea nozakii*

## Abstract

Background: Medusozoans utilize explosively discharging penetrant nematocysts to inject venom into prey. These venoms are composed of highly complex proteins and peptides with extensive bioactivities, as observed in vitro. Diverse enzymatic toxins have been putatively identified in the venom of jellyfish, *Nemopilema nomurai* and *Cyanea nozakii*, through examination of their proteomes and transcriptomes. However, functional examination of putative enzymatic components identified in proteomic approaches to elucidate potential bioactivities is critically needed. Methods: In this study, enzymatic toxins were functionally identified using a combined approach consisting of *in gel* zymography and liquid chromatography tandem mass spectrometry (LC-MS/MS). The potential roles of metalloproteinases and lipases in hemolytic activity were explored using specific inhibitors. Results: Zymography indicated that nematocyst venom possessed protease-, lipase- and hyaluronidase-class activities. Further, proteomic approaches using LC-MS/MS indicated sequence homology of proteolytic bands observed in zymography to extant zinc metalloproteinase-disintegrins and astacin metalloproteinases. Moreover, pre-incubation of the metalloproteinase inhibitor batimastat with *N*. *nomurai* nematocyst venom resulted in an approximate 62% reduction of hemolysis compared to venom exposed sheep erythrocytes, suggesting that metalloproteinases contribute to hemolytic activity. Additionally, species within the molecular mass range of 14–18 kDa exhibited both egg yolk and erythrocyte lytic activities in gel overlay assays. Conclusion: For the first time, our findings demonstrate the contribution of jellyfish venom metalloproteinase and suggest the involvement of lipase species to hemolytic activity. Investigations of this relationship will facilitate a better understanding of the constituents and toxicity of jellyfish venom.

## 1. Introduction

Jellyfish belong to the phylum Cnidaria, and comprise hydrozoans, scyphozoans and cubozoans [1,2]. Cnidae are characteristic features of all cnidarians, and also the means with which cnidarians capture prey and defend themselves. The number of jellyfish blooms occurring in the coastal areas of the world has been increasing in recent decades [3,4]. A negative consequence of this massive increase in jellyfish is the increasing number of jellyfish envenomations, one of the most commonly occurring marine envenomations. The symptoms induced by jellyfish stings vary depending on the jellyfish species. For instance, stings from a cubozoan, such as *Chironex fleckeri*, may induce rapid, life-threatening respiratory and cardiovascular arrest [5,6,7]. Jellyfish nematocyst venom, composed of highly complex and toxic mixtures of proteins and peptides, is the underlying basis of severe envenomations. To date, some biological activities of these proteinaceous mixtures have been observed, including hemolytic [8,9], insecticidal [10,11], cardiotoxic [12,13] and cytotoxic [14,15] activity.

The jellyfish *Nemopilema nomurai* and *Cyanea nozakii* are the major blooming species along the coast of China in the Yellow Sea. Although they are not as toxic as cubozoans, severe envenomations by these jellyfish are common, and the public concern regarding human health related to these envenomations in China’s coastal areas during the summer is increasing. Clinical manifestations include itching, swelling, acute pain, local erythrosis, and inflammation, and in severe cases, victims may die within hours [16]. However, the principal pathophysiological components and mechanisms of action have yet to be determined. Numerous efforts have been made to bridge this knowledge gap. Applications of proteomics and transcriptomics to elucidate jellyfish venom have suggested that many proteins in jellyfish venom exhibit notable sequence homologies with known enzymatic toxins, such as metalloproteinase and phospholipase A_2_ (PLA_2_) toxins, at the proteomic and transcriptomic levels [16,17]. In accordance with these proteomic and transcriptomic data, previous study demonstrated that *N. nomurai* and *C. nozakii* nematocyst venom possessed significant metalloproteinase and PLA_2_-like activities by biochemical and kinetic analysis [18]. Interestingly, metalloproteinases and PLA_2_s from other venomous animals, such as snakes and scorpions, have been found to mediate the toxic effects that occur after envenomation [19]. These findings suggest that toxin species with homology to known enzymes may contribute to the envenomation related pathogenic sequelae. However, while proteomic and transcriptomic data provide important information, direct experimental studies are needed to explore whether these enzymatic constituents play a functional role in jellyfish envenomation.

To explore the potential role of the enzymatic toxins in jellyfish venom cocktails in biological activities in vitro, we chose hemolytic activity, which is the most well characterized activity in jellyfish venom research, as a core functional assay. In this study, a liquid chromatography tandem mass spectrometry (LC-MS/MS) paired structure function study was performed to characterize bands obtained from *in gel* zymography. Specifically, nematocyst venoms from *N. nomurai* and *C. nozakii* were separated on non-reducing gels to assay for protease, lipase, and hyaluronidase as well as cytolytic constituents. Additionally, class specific inhibitors were used to begin to clarify the precise biochemical activities of species with sequence homologies to metalloproteinases, PLA_2_. Our findings suggest that diverse functional proteases were included in jellyfish nematocyst venom, and they were putatively identified as metalloproteinases. Moreover, the metalloproteinases and PLA_2_-specific inhibitor sensitive species were found to contribute to the hemolytic activity of jellyfish venom by employing selective inhibitors, batimastat and varespladib.

## 2. Results and Discussion

### 2.1. Evaluation of the Molecular Mass of the Enzymatic Components by Zymography Assays

**Figure 1:**
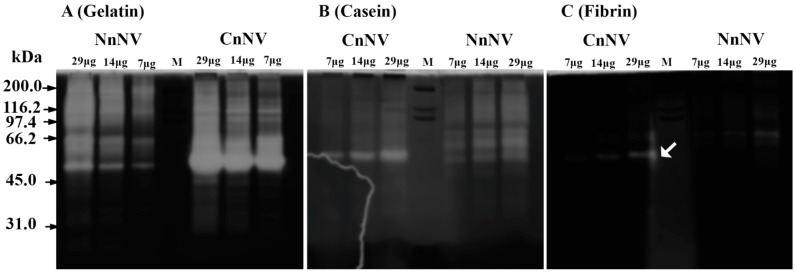
Zymograms of proteolytic enzymes in jellyfish nematocyst venom. In the protease zymograms, three different substrates: gelatin (0.2% *w*/*v*) (**A**); casein (0.2% *w*/*v*) (**B**); and fibrin (0.12% *w*/*v*) (**C**), were used and the protease activity appeared as clear bands or regions. White arrow indicates that only CnNV exhibited fibrinolytic activity at ~57 kDa and NnNV exhibited slight activity at ~66 kDa. M, marker.

Figure 1 shows that *N*. *nomurai* nematocyst venom (NnNV) and *C*. *nozakii* nematocyst venom (CnNV) utilized gelatin, casein and fibrin as substrates in a venom-concentration dependent manner. In addition, various zymolytic band patterns were detected in the zymograms of proteolytic enzymes, which imply variations between the enzymatic components of NnNV and CnNV. More activity was detected with the substrate gelatin than casein (Figure 1A). In gelatin zymogram, the intensity of the band with a molecular weight of ~57 kDa was the highest among all detected zymolytic bands, even when the substrate gel was loaded with ~7 μg of CnNV protein. In contrast to gelatin zymogram, fewer bands emerged for caseinolytic activity, and the intensities of the zymolytic bands were markedly decreased (Figure 1B). Comparative zymography using casein as the substrate, showed less activity in CnNV samples than that in NnNV. In Figure 1C, both NnNV and CnNV showed weak proteolytic activity toward 0.12% (*w*/*v*) fibrin at a venom concentration range of 7 μg~29 μg. CnNV exhibited fibrinolytic activity at about 57 kDa while NnNV exhibited slight activity at ~66 kDa.

The zymograms of the venom revealed a high abundance of gelatinases, caseinolytic enzymes and fibrinases in NnNV and CnNV, most of which possessed relatively high molecular masses ranging from ~45 kDa to ~200 kDa. The current findings are consistent with the findings of previous reports [20] and highlight the proteases with high molecular masses.

**Figure 2:**
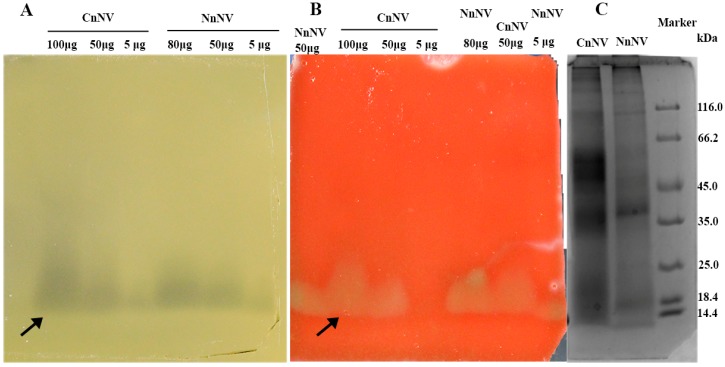
(**A**) lecithinolytic activity of *N*. *nomurai* nematocyst venom (NnNV) and *C*. *nozakii* nematocyst venom (CnNV). The lecithinolytic activity was assayed using egg yolk as substrate. After electrophoresis under non-reducing conditions, the resolving gel was further overlaid onto the substrate gel containing egg yolk lecithin, instead of staining with Coomassie blue R-250, to allow the diffusion of lipase-class enzymes into the substrate gel and then incubated at 37 °C to visualize the transparent zone against the yellow background. (**B**) Hemolysis zymogram on a gel containing 10% egg yolk and 3% sheep erythrocytes. This hemolysis zymogram was used to analyze the possible involvement of lipase-class enzymes in NnNV and CnNV in hemolytic activity. Arrows indicate the lecithinolytic activity or hemolysis. (**C**) SDS-PAGE profiles of NnNV and CnNV under non-reducing conditions and stained with 0.25% Coomassie blue R-250.

A venom-concentration dependent zone of egg yolk lysis was observed in Figure 2A, in both NnNV and CnNV samples with an approximate molecular mass of 14–18 kDa. Egg yolk is not a specific substrate for PLA_2_, and it can be hydrolyzed by many types of lipases, including phospholipases such as phospholipase A (PLA) and phospholipase C (PLC) [21], so the nature of the protein responsible for egg yolk lysis remains unknown. Based upon the molecular mass and previous published findings, another possible contributor to egg yolk hydrolysis is the small (13~19 kDa) secretory PLA_2_ class lipases. PLA_2_-like activity has been extensively reported using ^14^C-labelled arachidonic acid in the sn-2 position or 4-nitro-3-octanoyloxybenzoic acid as substrates in various cnidarians, including *Cyanea capillata*, *Cyanea lamarckii* (Pe′ron and Le′slieur) and *Olindias sambaquiensis* [22,23,24]. Moreover, when sheep erythrocytes were added into the substrate gel, marked hemolysis occurred at the location where the lipase hydrolyzed the egg yolk substrate (Figure 2B).

**Figure 3:**
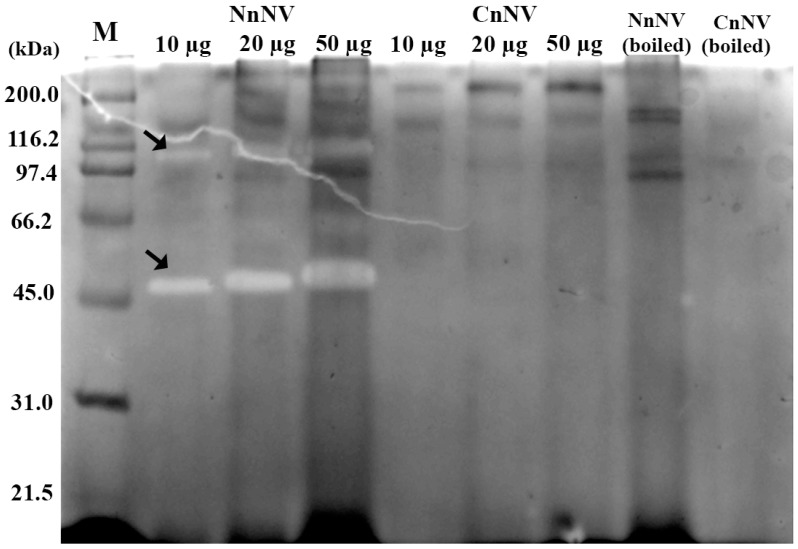
Zymogram of hyaluronidase. In hyaluronidase zymogram, 0.17 mg/mL hyaluronic acid was incorporated into the resolving gel for electrophoresis together with 10–50 μg of NnNV or CnNV under non-reducing conditions. The substrate gel was stained with 0.1% alcian blue and the presence of clear translucent bands against the dark blue background reveals the existence of hyaluronidase. CnNV was found incapable of degrading hyaluronic acid under the experimental conditions. In addition, as negative controls, 20 μg of NnNV and CnNV were boiled for 10 min at 100 °C to inactivate the hyaluronidases. Arrows indicate the existence of hyaluronidases. M, markers.

Hyaluronic acid zymogram analysis further revealed two molecular weight species (~48 kDa and ~105 kDa) exhibiting concentration-dependent lysis of hyaluronic acid (Figure 3). However, no hyaluronidase activity was detected in CnNV hyaluronidase zymogram. These current findings are, however, contradictory to a previous study in which *N. nomurai* venom was unable to degrade hyaluronic acid [20]. This discrepancy, we think, may partially result from the venom variation and nematocyst purity used for venom extraction.

### 2.2. Venom Protein Profiling by LC-MS/MS

**Figure 4:**
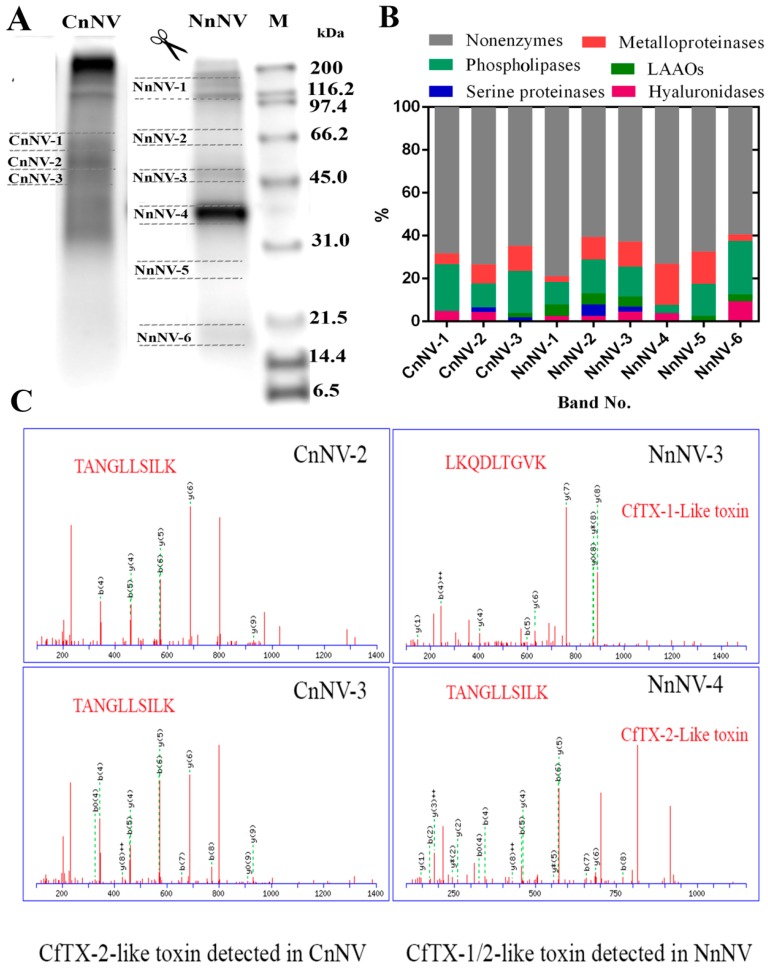
(**A**) SDS-PAGE profile of NnNV and CnNV under non-reducing conditions. The resulting gels were excised into nine sections, as indicated by the dashed line. These sections demonstrated protease and phospholipase activities in zymography. (**B**) Overall view of the LC-MS/MS identification results of enzymatic and non-enzymatic components in the digests of NnNV and CnNV band slices. The distribution and proportions (%) of potential enzymatic components in different band slices of NnNV and CnNV are indicated by different colors. (**C**) Representative MS^2^ spectrum and corresponding sequences of peptide fragments from CfTX-1 or CfTX-2 identified in tryptic peptides of NnNV-3–NnNV-4 or CnNV-2–CnNV-3.

To investigate the structural basis of the function observed in the zymography assays, LC-MS/MS analysis of tryptic peptides was performed after one-dimensional SDS-PAGE separation of jellyfish nematocyst venom under non-reducing conditions. Gel slices (*n* = 9) bearing different enzymes according to the zymography assays were excised as shown in Figure 4A. Figure 4B shows an overview of the results of the proteomic identification of the potential enzymatic and non-enzymatic toxins obtained by searching tryptic peptides in the UniProt animal venom and toxins database [25]. The putative enzymes identified from tryptic digests of NnNV and CnNV (45–66 kDa) are listed in Tables S1 and S2. The potential proteases responsible for the proteolytic activity observed in the zymography assays showed some homology to zinc metalloproteinase-disintegrin-like and astacin-like metalloproteinases. Disintegrin-like and astacin-like metalloproteinases have been suggested in scyphozoan [16,17,26], cubozoan [27] and hydrozoan [28,29] jellyfish venom, which indicates the extensive distribution of these toxin families among cnidarians.

Interestingly, one potential toxin metalloproteinase identified in CnNV-2 (~55 kDa) was homologous with zinc metalloproteinase-disintegrin-like VAP2A from *Crotalus atrox*, which has a molecular mass of approximately 55 kDa in the absence of a reducing regent [30]. Since CnNV showed significant proteolytic activity at ~57 kDa, this homologue was strongly suggested to have contributed to the proteolysis observed in the protease zymograms (Figure 1). Moreover, putative metalloproteinases that were homologous with zinc metalloproteinase carinactivase-1 (Q9PRP9) from *Echis carinatus* and astacin-like metalloprotease toxin 1 (A0FKN6) from *Loxosceles intermedia*, which had previously been suggested in the *N. nomurai* proteome [17], were also detected at 35–46 kDa, corresponding to the gel bands NnNV-2–NnNV-4 (Table S2). Combining the protease zymograms with LC-MS/MS identification suggested a group of active metalloproteinases in scyphozoan venom, but the biological significance of the abundant metalloproteinases in the stinging nematocysts remains unclear. The metalloproteinases in jellyfish nematocyst venom might serve to digest prey through their proteolytic effects on various protein substrates or might function as toxins. Snake venom metalloproteinases (SVMPs) are multifunctional proteins and important sources of new enzymes with therapeutic potential. In this study, various proteases with relatively high molecular masses were observed in zymography assays, and are in contrast to the lower molecular weight rattlesnake venom metalloproteinases species (e.g., 19–37 kDa and 53 kDa) [31]. Therefore, the high abundance of metalloproteinases in jellyfish venom may provide a new source of novel metalloproteinases.

In addition to the lipase-class enzymes indicated by zymography (Figure 2), other lipases, including the phospholipases D (PLD) and phospholipase A_1_ (PLA_1_), as suggested at the proteomic level, are potential constituents of jellyfish nematocyst venom (Tables S1 and S2). PLD, known as dermonecrotic toxin, is the most well characterized toxin in spider venom and is mainly responsible for local and systemic spider-bite-induced symptoms, including dermonecrosis and intense inflammatory reactions [32,33]. Of note, the PLD family has been identified in the *C. capillata* transcriptome [34], and the present study provides the first indication that PLD-like toxins might exist in NnNV and CnNV at the proteomic level.

Hyaluronidases in snake venom are known to be an important group of enzymes that degrade hyaluronic acid in the extracellular matrix of local tissues and therefore facilitate the spreading of venom in prey or humans after a snake bite [35]. In this study, hyaluronidases were identified functionally by *in gel* zymography as well as using proteomic approaches in *N. nomurai* venom for the first time. The distribution of these enzymes in the gel slices was much lower than those of the metalloproteinases and phospholipases (Figure 4B). This result agrees with the zymogram of hyaluronidase, which only showed two transparent bands against the blue background above 45 kDa (Figure 3).

Apart from the metalloproteinases, lipases and hyaluronidases revealed in the zymography assays, other toxin enzymes, such as serine proteases and L-amino acid oxidases (LAAOs), were also detected in NnNV and CnNV (Figure 4B). Increasing number of reports focus on the experimental characterization of toxin enzymes in jellyfish venom. The functional activity and cDNA sequence of *N. nomurai* chymotrypsin protease has recently been reported [36]. Moreover, nematocyst venom from the hydrozoan jellyfish *O*. *sambaquiensis* was also observed to have considerable serine protease activity, which was comparable to that of *Bothrops* snakes venom [24].

### 2.3. Hemolytic Activity and the Roles of Metalloproteinase- and Lipase-Class Species in Hemolysis

**Figure 5:**
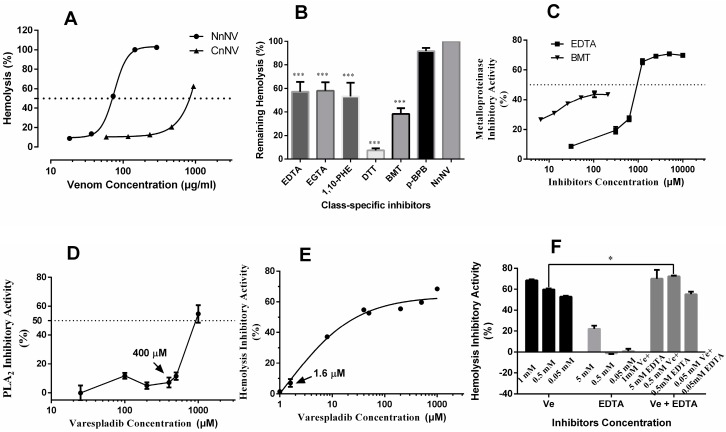
(**A**) Hemolytic activity of NnNV and CnNV assayed with 1% sheep erythrocytes. The error bars are within the symbol. The dotted line represents 50% hemolysis of 1% sheep erythrocytes. (**B**) Effects of class-specific inhibitors on hemolytic activity. EDTA (3.85 mM, final concentration), EGTA (3.85 mM), 1,10-PHE (3.85mM), DTT (3.85 mM), BMT (40 μM), and *p*-BPB (2.5 mM) were pretreated with NnNV for 30 min at 37 °C, and the residual hemolytic activity was then measured. (**C**) Effects of metalloproteinase inhibitors, EDTA and BMT, on the metalloproteinase activity of NnNV. Assays were conducted using a common protease substrate azocasein. (**D**) Effect of PLA_2_ inhibitor, varespladib, on the phospholipases activity of NnNV assayed with a chromogenic substrate NOBA. The inhibitory effects of: varespladib (**E**); or varespladib in combination with EDTA (**F**) at different concentrations on hemolytic activity of NnNV. All the results are expressed as the mean ± S.E.M. and at least three replicates were set for each assay. * *p* < 0.05 and *** *p* < 0.001 are considered significant. Abbreviations: EDTA, ethylenediaminetetraacetic acid; BMT, batimastat; EGTA, ethylene glycol-bis(β-aminoethyl ether)-*N*,*N*,*N*’,*N*’-tetraacetic acid; 1,10-PHE, 1,10-phenanthroline; DTT, dithiothreitol; Ve, varespladib.

The hemolytic effects of NnNV and CnNV were assessed using a sheep erythrocyte suspension. Figure 5A shows that NnNV, with an HU_50_ value equal to 75.4 ± 1.0 μg protein/mL (*n* = 3, *R*^2^ = 0.9989), was more hemolytic than CnNV toward sheep erythrocytes. However, the HU_50_ value of CnNV was not determined because even high concentration of CnNV (protein concentration, 937.5 μg/mL) only induced 62.5% ± 0.5% (*n* = 3) erythrocyte lysis (Figure 3A).

The potential roles of NnNV metalloproteinases and lipase-class enzymes in hemolytic activity were examined for the first time in this study. Although extensive hemolysis was observed in jellyfish venom extracted from the whole tentacle tissues or isolated nematocysts, the chemical nature of the toxins underlying the hemolytic effects was unclear. Previous studies have suggested that cytolysins or pore-forming toxins, such as CfTX-1/2, CaTX-A, CrTXs and CqTX-A, possess a highly conserved transmembrane spanning region (TSR1) and might act through a pore-forming mechanism to exert hemolytic effects [37,38,39]. The correlation between hemolytic activity and the enzymatic properties of jellyfish venom has rarely been investigated. In an earlier study, crude venom from the jellyfish *Carybdea alata*, now classified as *Alatina alata* [40,41], that was incubated with 20 mM of ethylenediaminetetraacetic acid (EDTA) lost all its hemolytic activity, and the authors addressed the importance of divalent ions, such as Ca^2+^ and Mg^2+^, in hemolytic activity [42]. The effects of divalent ions on hemolytic effects have been extensively investigated and found to vary depending on the jellyfish species. Among these ions, metal ions, such as Cu^2+^ and Mn^2+^, have been reported to inhibit the hemolytic activity of jellyfish venom, whereas determination of the effects of other divalent ions, such as Ca^2+^, Mg^2+^, Sr^2+^, and Ba^2+^ was inconclusive, with varying effects depending on the jellyfish species and ion concentration [8,42,43]. Ca^2+^ was found to increase hemolytic activity in a concentration-dependent manner below 1 mM, while appearing to exert no influence at relatively higher concentrations (1–2 mM and 10 mM). Mg^2+^, Sr^2+^, and Ba^2+^ were found to have similar effects on the hemolytic activity of *C. nozakii* venom, while 10 mM Mg^2+^ and Ba^2+^ inhibited the hemolytic activity of *Pelagia noctiluc*a venom to different extents. Whether divalent ions modulated hemolytic activity by influencing the enzymatic activity of jellyfish venom remains unknown. To investigate the potential roles of metalloproteinases and PLA_2_-like toxins in hemolytic activity in vitro, NnNV was incubated with metalloproteinase inhibitors, EDTA, EGTA, 1,10-phenanthroline and batimastat, or PLA_2_ inhibitors, *p*-bromophenacyl bromide and varespladib, at 37 °C for 30 min, and the hemolytic activity was measured. Metalloproteinase inhibitors, EDTA, EGTA and 1,10-phenanthroline [44], significantly protected sheep erythrocytes from lysis (Figure 5B), which suggests that enzymatic components, such as metalloproteinases and PLA_2_-like toxins, might contribute to the hemolytic effects towards sheep erythrocytes. Because metalloproteinase inhibitors, such as EDTA, inhibited metalloproteinases, PLA_2_s [45,46] as well as many other potential venom components such as pore forming toxins, a highly specific metalloproteinase inhibitor, batimastat, was used. When NnNV was pre-incubated with 40 μM of batimastat, only 38.3% ± 2.4% (*n* = 3) lysis occurred, which was significantly lower than that observed in the untreated venom (*p* < 0.0001) and with other metalloproteinase inhibitors (*p* < 0.05). Moreover, EDTA and batimastat were found to concentration-dependently inhibit NnNV metalloproteinases, and batimastat is observed to have higher inhibitory efficiency than EDTA below 200 μM (Figure 5C). Although the biofunctional role of the high abundance of metalloproteinases in scyphozoan venom remains elusive, the current findings indicate that metalloproteinases might be involved in hemolysis in vitro. Studies have revealed that metalloproteinases in snake venom mediate various pathological symptoms, including hemorrhage, edema, inflammation, and muscle necrosis [47]. Previous studies have also demonstrated that scyphozoan jellyfish metalloproteinases might be involved in cytotoxicity [20] and severe TE-induced liver and kidney hemorrhagic injuries [48]. These observations highlight the multi-functionality of metalloproteinases from various animal toxins. To further understand the medical relevance of the enzymatic components of jellyfish envenomations, much work is needed, including the identification and separation of additional jellyfish metalloproteinases.

As reported, the PLA_2_ inhibitor *p*-bromophenacyl bromide (*p*-BPB) can modify the His48 residue, which is conserved in PLA_2_s and is essential for enzymatic activity [49]. However, in this study NnNV modified by *p*-BPB still exhibited considerable hemolysis (Figure 5B). To determine the potential involvement of secretory PLA_2_ in hemolytic activity, a specific secretory PLA_2_ inhibitor, varespladib [50], is used in this study. Figure 5D shows that varespladib exhibited concentration-dependent inhibition against NnNV lipases from 400 μM to 1000 μM, however the inhibitory effect was almost negligible below 400 μM. The effect of varespladib to inhibit hemolysis was also examined. Potent inhibition of sheep erythrocyte hemolysis was observed after venom pre-incubation with very low concentrations of varespladib (Figure 5E). The minimum concentration was 1.6 μM. This finding is most remarkable and unprecedented. In phospholipase inhibition assay, the specific PLA_2_ substrate, chromogenic 4-nitro-3-octanoyloxybenzoic acid (NOBA) was used [51,52,53], to provide clarification to the egg yolk zymography data since egg yolk may be hydrolyzed by many types of lipases. NOBA was originally used to measure the PLA_2_ activity in snake venom research, however this chromogenic substrate was not very specific to PLA_2_ because PLA1 and phospholipase B (PLB) also utilize NOBA as substrate [54]. The phenomenon that relatively high and presumably non-specific concentrations of varespladib (>400 μM) were needed in phospholipase inhibition assay may be explained by the possible involvement of other phospholipases in degrading the substrate NOBA. Alternatively, the venom PLA_2_ exhibits differential sensitivity to this specific inhibitor. The concentration difference that varespladib shows in inhibiting phospholipase activity and hemolytic activity of NnNV may also indicate the key role of PLA_2_-specific inhibitor sensitive species in NnNV-induced hemolysis. Whether this PLA_2_-specific inhibitor sensitive species is a member of the secretory PLA_2_ family, porins or other yet to be determined proteins requires further investigation. Interestingly, we observed that varespladib in combination of different concentrations of EDTA significantly inhibited the hemolysis while EDTA (<0.5 mM) alone failed to exhibit any hemolysis inhibitory effects (Figure 5F). In fact, the hemolytic effects of many PLA_2_s from other venomous animals, including snakes, corals, and scorpions, have been extensively documented [55,56,57]. However, PLA_2_s that lack hemolytic activity were also found in the nematocyst venom of a sea anemone [46,58]. These findings highlight the variations and versatility of PLA_2_s from different venomous species. Jellyfish and sea anemones constitute a sister group of the Cnidaria. However, no PLA_2_s had been previously isolated from jellyfish venom and their biological activities were still unknown. The observation that *N*. *nomurai* PLA_2_-like toxins are hemolytic underlines the necessity to investigate the PLA_2_ in jellyfish nematocyst venom.

Despite the importance of the metalloproteinases and PLA_2_-like toxins in jellyfish venom in terms of hemolytic effects, the roles of non-enzymatic components or other enzymatic constituents should not be neglected considering the considerable hemolysis observed after the inhibition of NnNV by EDTA, ethylene glycol-bis(β-aminoethyl ether)-*N*,*N*,*N*’,*N*’-tetraacetic acid (EGTA), batimastat and varespladib (Figure 5). This remaining hemolysis might be attributed to other non-enzymatic components, such as cytolysins. According to earlier reports, the molecular weights of the CfTX toxin family, including CaTX, CrTX, and CqTX, were all approximately 45 kDa, as estimated by SDS-PAGE [37,39,59,60,61]. Accordingly, the CfTX toxin family was exclusively found in the gel bands of CnNV (~45–55 kDa) and NnNV (~35–46 kDa) (Table S3, Figure 4C). Although the proteomic data used for the identification of CfTX toxins were not of high quality, this result was unexpected because previous transcriptomic and proteomic analyses of venom from the jellyfish *S*. *meleagris* did not reveal any members of this toxin family [17,62]. However, in the tentacle transcriptome of the jellyfish *C*. *capillata*, researchers identified a transcript (Unigene6213) with a predicted transmembrane spanning region (TSR1) that had high similarity to other cnidarian cytolysins [34]. Moreover, increasing evidence has revealed that homologues of the CfTX toxin family are not confined to cubozoan nematocyst venom but can also be detected in the proteomes of scyphozoan nematocyst venom, including the jellyfish *C*. *capillata* [34], *Aurelia aurita* [63], *Chrysaora fuscescens* [26] and *Chrysaora quinquecirrha* [61]. The current observations support the idea that members of the CfTX-like toxin family could be constituents of NnNV and CnNV, although their abundance was not high as that in the box jellyfish *C. fleckeri*.

## 3. Conclusions 

In conclusion, LC-MS/MS identification data further elucidate the functional basis of jellyfish venom on various substrates in zymography assays and an emerging picture suggests that metalloproteinases, lipases and hyaluronidases are key components. Moreover, specific inhibitors, batimastat and varespladib, were found to inhibit metalloproteinases and lipolytic enzymes in jellyfish venom, which strongly suggest the provisional identities assigned by LC-MS/MS data. However, these identity assignments are still tentative because we found the concentration range of varespladib required for PLA_2_ inhibition was far higher than that required for snake secretory PLA_2_. These results may indicate the possible involvement of other types of lipases, such as PLD and PLA_1_, in catalytic hydrolysis of egg yolk or NOBA by NnNV. Interestingly, when varespladib was used in hemolytic assays, a significant loss of the hemolytic effects of NnNV was observed at micromolar concentrations. Thus, PLA_2_-specific inhibitor sensitive species may be suggested as the key hemolysis-inducing component in NnNV. Further, direct evidence that NnNV lipases (or another yet to be determined varespladib-sensitive toxin), induce egg yolk lysis and sheep erythrocytes hemolysis with molecular masses of 14–18 kDa was demonstrated. NnNV PLA_2_ may only exhibit limited catalytic activity yet still insert into RBC lipid bilayer and effect hemolysis. Future studies are merited to clarify the role of NnNV PLA_2_ and other lipase species. NnNV metalloproteinases were also found to contribute to the hemolytic effects of NnNV by employing a panel of metalloproteinase inhibitors, batimastat, EDTA and 1,10-phenanthroline. These data provide key clues in the elucidation of mechanistic basis of jellyfish venom induced biological activities and will lead to potential therapeutic targets for treating jellyfish stings.

Although we sought to elucidate the enzymatic constituents of scyphozoan venom and their potential functional roles in hemolytic activity, these findings demonstrate the need for as well as inform additional studies to further elucidate the key toxins involved. LC-MS/MS identification of tryptic peptides offers limited information on the identities of enzymatic components in jellyfish venom, so functional and inhibitor specific effects-guided isolation of specific enzymes, such as metalloproteinase and PLA_2_, should be conducted in the near future.

## 4. Materials and Methods 

### 4.1. Samples

All the jellyfish species used in this study, *N. nomurai* and *C. nozakii*, were collected in the village of Huangshan in Qingdao, Shandong Province, China, in August 2013. After capture, the fishing tentacles of both scyphozoans were excised immediately and pooled in plastic zip-pack bags for storage at −80 °C. In China, the coastal waters usually gather numerous jellyfish during the summer, and the number of jellyfish has been increasing [3]. Because jellyfish are not endangered or protected in China, fishing for jellyfish is permitted by the department of fisheries.

### 4.2. Isolation of Nematocysts and Venom Extraction

The fishing tentacles of jellyfish were used to isolate the nematocysts, as described in the literature [64,65] with a slight modification. Briefly, the frozen tentacles were taken out from −80 °C freezer and thawed in seawater for one day to allow the autolysis of the tentacles at 4 °C. On the second day, the supernatant of the mixtures was gently poured out, and fresh natural seawater was added. This procedure was repeated for several days. Then, the remaining tentacle tissue was removed by a 200-mesh plankton net. The filtrate was collected and then subject to centrifugation at 1500× *g* for 10 min at 4 °C. Nematocysts were further obtained by collecting the resulting pellet. Venom preparation was conducted from the resulting nematocysts using the methods described by Rongfeng Li [66]. Briefly, the nematocysts were suspended in cold NEB (nematocyst extraction buffer, 20 mM ${PO}_{4}^{3-}$, 150 mM NaCl, pH 7.4) and extracted employing Ultra-Turrax JY92-II (Scientz, Ningbo, China) at 400 W for 90 cycles. The extraction condition was set as follows: each cycle contains 10 s of sonication and 15 s of rest on ice. The venom extracts were pooled and centrifuged at 15,000× *g* for 20 min at 4 °C. The protein concentration of both venom samples was determined using Folin-Phenol reagent (DingGuo ChangSheng Biotechnology Co. Ltd., Beijing, China) according to the manufacturer’s instructions.

### 4.3. SDS-PAGE Analysis

Twelve percent SDS-PAGE electrophoresis was performed according to Laemmli’s method [67] under non-reducing conditions. Briefly, under non-reducing conditions, 40 μL of NnNV (1.45 μg/μL) or CnNV (1.87 μg/μL) were treated with 10 μL of non-reducing 5 × sample buffer (0.5 M Tris-HCl, pH 6.8, 50% (*v*/*v*); SDS, 100 mg/mL; glycerol, 30% (*v*/*v*); bromophenol blue, 0.5 mg/mL). These samples were incubated at room temperature for 5 min in the absence of heat (100 °C for 10 min) and a reducing agent (β-mercaptoethanol). Then, approximately 30 μg of protein from NnNV, CnNV and 10 μL of molecular weight standards were loaded onto Mini-PROTEAN^®^ TGX™ gels (Bio-Rad, Hercules, CA, USA) and run in electrophoresis buffer containing 25 mM Tris, 192 mM glycine and 1% SDS under non-reducing conditions. Electrophoresis was carried out at 120 V for approximately 90 min at 4 °C using a Mini-PROTEAN Tetra apparatus (Bio-Rad, Hercules, CA, USA). After electrophoresis, the protein bands were visualized by staining with 0.25% Coomassie Brilliant Blue R-250. The following molecular weight standards were used in this study: Bio-Rad broad range standards (aprotinin, 6.5 kDa; lysozyme, 14.4 kDa; trypsin inhibitor, 21.5 kDa; carbonic anhydrase, 31.0 kDa; ovalbumin, 45.0 kDa; serum albumin, 66.2 kDa; phosphorylase b, 97.4 kDa; β-galactosidase, 116.25 kDa; and myosin, 200.0 kDa) (Bio-Rad, Hercules, CA, USA) or unstained protein marker (lysozme, 14.4 kDa; β-lactoglobulin, 18.4 kDa; REase Bsp98l, 25.0 kDa; lactate dehydrogenase, 35.0 kDa; ovalbumin, 45.0 kDa; bovine serum albumin, 66.2 kDa; β-galactosidase, 116.0 kDa) (Thermo Scientific, Waltham, MA, USA).

### 4.4. In Gel Zymography of Proteases, Lipases and Hyaluronidases

#### 4.4.1. Zymography of Proteases

The proteolytic enzymes in jellyfish venom were examined using zymography methods [68]. Briefly, the substrates gelatin and casein were polymerized into 12% SDS-PAGE gels at a final concentration of 0.2% (*w*/*v*). The cross-linked fibrin gel was prepared by incorporating 100 μL of thrombin (Sigma, St. Louis, MO, USA, 10 NIH/mL) into an SDS-PAGE gel matrix containing 0.12% (*w*/*v*) fibrinogen from human plasma and polymerized overnight [69]. These substrate gels were loaded with various amounts of NnNV or CnNV and run at 120 V and 4 °C for approximately 120 min under non-reducing conditions. The gels were then washed twice for 40 min in 2.5% Triton X-100 followed by washing twice for 30 min in assay buffer (50 mM Tris-HCl, 200 mM NaCl, 5 mM CaCl_2_, pH 8.8). After incubating in assay buffer for 24 h, the substrate gels were stained with 0.25% Coomassie blue R-250 and then destained with a methanol:glacial acetic acid:distilled water (5:1:4) solution.

#### 4.4.2. Zymography of Lipases

The molecular weights of the PLA_2_s-like toxins in jellyfish venom were evaluated by zymography of lipases [70]. Briefly, various amounts of NnNV and CnNV were electrophoresed at 120 V and 4 °C on a 12% polyacrylamide gel under non-reducing conditions. The gel was washed twice for 40 min in 50 mM Tris-HCl, pH 7.6, containing 2.5% (*v*/*v*) Triton X-100. Then, the gel was washed twice for 30 min in 50 mM Tris-HCl, 100 mM NaCl and 5 mM CaCl_2_ (pH 8.0). The gel was then incubated for 12 h at 37 °C over a 1.0% (*w*/*v*) agarose gel prepared in 50 mM Tris-HCl, 100 mM NaCl and 5 mM CaCl_2_ (pH 8.0) and 10.0% egg yolk. Clear zones against the yellow background may indicate the presence of lipases in the jellyfish venom.

#### 4.4.3. Zymography of Hemolytic Proteins 

To detect the hemolytic components in jellyfish venom, a modified zymography method was used according to the literature [71]. Briefly, various amounts of NnNV or CnNV was run (120 V, 4 °C, 120 min) under non-reducing conditions. The gel was then washed twice for 30 min with 20 mM Tris-HCl (pH 7.4) containing 2.5% (*v*/*v*) Triton X-100. Then, the gel was further washed twice for 30 min in 50 mM Tris-HCl, 100 mM NaCl and 5 mM CaCl_2_ (pH 8.0), to remove the SDS and Triton X-100. After that, the gel was overlaid on a 1% agarose gel containing 10.0% egg yolk and 3% PBS-washed sheep erythrocytes in 20 mM Tris-HCl, 5 mM CaCl_2_ (pH 7.4). After incubating the gel for 12 h at 37 °C, the presence of clear bands in the agarose gel indicated the existence of hemolysins.

#### 4.4.4. Zymography of Hyaluronidases

Hyaluronidases in jellyfish venom were visualized by zymography as described by [72] with slight modification. Briefly, the gel for electrophoresis was prepared by incorporating hyaluronic acid at a final concentration of 0.17 mg/mL into a 12% SDS-PAGE gel matrix. Then, the substrate gel was loaded with different concentration of NnNV or CnNV and run at 120 V and 4 °C for approximately 120 min under non-reducing conditions. After electrophoresis, the gels were washed twice with 2.5% Triton X-100 for 40 min to remove SDS and incubated twice for 30 min in the assay buffer (100 mM sodium acetate buffer containing 150 mM NaCl, pH 6.0) to remove the Triton X-100. After incubation for 24 h at 37 °C in the assay buffer, the gel was then stained with 0.1% alcian blue for 3 h and destained in 5% acetic acid until clear translucent bands appeared against the dark blue background.

### 4.5. Protein Identification by LC-MS/MS

Venom proteins were separated using 12% SDS-PAGE as described in Section 4.3 under non-reducing conditions and stained with colloidal Coomassie Blue R-250. The resulting gel was cut into pieces according to the protease or lipase zymograms. Nine pieces of gel were obtained: 3 from CnNV (~45–66 kDa) and 6 from NnNV. Then, the venom proteins were digested in the gel and extracted as follows: each gel band was placed in 1.5 mL tubes, and de-stained twice for 10 min with 50% acetonitrile and 25 mM ammonium bicarbonate, and then dehydrated with 100% acetonitrile. After removing the solvent, the gel bands were reduced and alkylated with 10 mM dithiothreitol and 55 mM iodoacetamide, respectively. Then, the samples were dehydrated with neat acetonitrile and dried under reduced pressure. A trypsin solution (1 μg/μL) was diluted in 25 mM ammonium bicarbonate and then added for digestion. Mixtures containing trypsin and the samples were incubated overnight at 37 °C. The extracted tryptic peptides were then run on a MicrOTOF-Q II mass spectrometer (Bruker Daltonics, Billerica, MA, USA) for analysis. Briefly, 10 μL of tryptic peptides was separated using a prominence nano 2D HPLC system (Shimadzu, Kyoto, Japan) equipped with a C18 reversed-phase column (5 μm, 150 Å, Eprogen). The mobile phase consisted of solvent A (0.1% *v*/*v* formic acid) and solvent B (100% acetonitrile in 0.1% formic acid *v*/*v*) and the separation conditions were set as follows: 0–4 min, 5% B; 5–30 min, 5%–40% B; and 31–35 min, 40%–80% B. The eluted peptides were analyzed in positive ion mode, and the capillary voltage was set at 1500 V. The full scan ranged from *m*/*z* 50 to 2000, and the 20 most-intense peptide ions were automatically selected for tandem mass spectrometry using collision-induced fragmentation in the linear ion trap. The Mascot search engine version 2.3.01 (Matrix Science, London, UK) was used for database searching against an online Swiss-Prot toxin database (http://www.uniprot.org/program/Toxins).

### 4.6. Hemolytic Activity In Vitro

The hemolytic activity of crude NnNV and CnNV was determined using a sheep erythrocyte microplate assay described in the previous literature [73]. Heparinized sheep blood (Penlish Technology Co. Ltd., Beijing, China, batch number, 201601011) was centrifuged (3000× *g*, 4 °C, 10 min), and the pellet erythrocytes were washed three times in sterile phosphate-buffered saline (PBS, 20 mM ${PO}_{4}^{3-}$, 150 mM NaCl, pH 7.4) and centrifuged (3000× *g*, 4 °C, 10 min). The erythrocytes were diluted in PBS and adjusted to set the absorbance at 540 nm equal to 0.8~1.0 when 100% hemolysis occurred. Aliquots (200 μL) of PBS-diluted erythrocytes were added to 50 μL of 2-fold diluted samples of NnNV in quadruplicate in 1.5 mL microcentrifuge tubes on ice and then incubated at 37 °C for 30 min. The samples were chilled on ice for 5 min and centrifuged at 3000× *g* for 5 min at room temperature, and the supernatants (200 μL) were transferred to a 96-well plate. The absorbance of released hemoglobin was measured at 540 nm. References included 1% Triton X-100 in PBS and PBS alone to represent 100% and 0% lysis, respectively. Hemolysis rates were calculated as the percentage relative to complete lysis. HU_50_ values, defined as the concentration of protein that causes 50% lysis of sheep erythrocytes, were determined for the crude venom. The concentration–response curves of hemolysis were each fitted with a four-parameter logistic curve in GraphPad Prism 6.0 (GraphPad software, San Diego, CA, USA).

The potential roles of metalloproteinases and lipases in hemolytic activity were revealed by pre-incubation of NnNV with class-specific inhibitors (3.85 mM EGTA, 3.85 mM EDTA, 3.85 mM 1,10-PHE, 40 μM BMT, 3.85 mM DTT, 2.5 mM *p*-BPB) for 30 min at 37 °C. To determine the effect of varespladib on the hemolysis induced by NnNV, varying concentrations of varespladib (final concentration, 0.016 μM–1000 μM) was preincubated with NnNV, then 200 μL of sheep erythrocyte was added and the hemolysis was measured as described above. In addition, the effect of varespladib in combination with EDTA on hemolysis was also determined. The relative hemolysis rate of NnNV that had been pre-incubated with inhibitors was calculated as the percentage relative to the hemolysis induced by NnNV in the absence of inhibitors.

### 4.7. Metalloproteinase Inhibitory Activity

The effects of metalloproteinase inhibitors, EDTA and batimastat, on the metalloproteinase activity of jellyfish venom were measured with azocasein [74]. Briefly, 100 μL of the reaction mixture contained 10 μL of EDTA (final concentration, 31.2 μM–10000 μM) or batimastat (final concentration, 6.5 μM–209.4 μM), 10 μL of NnNV (3.16 μg/μL) and 80 μL of azocasein (5 mg/mL) resuspended in assay buffer (50 mM Tris, 100 mM NaCl, 5 mM CaCl_2_, pH 8.8). The reaction mixture was then allowed for reaction at 37 °C for 90 min. Then, the assay was terminated by addition of 200 μL of 5% trichloroacetic acid at room temperature and centrifuged at 15,000× *g* for 15 min. After centrifugation, 150 μL of the supernatant was added into a 96-well plate and same volume of 0.5 M NaOH was also added into each well. Then, the absorbance was measured at 450 nm using an Infinite M100 plate reader (Tecan Group Ltd., Männedorf, Switzerland). A negative control was set to represent the inhibition of 0% by adding 10 μL of assay buffer into the reaction mixtures. Assays were performed in triplicate and the metalloproteinase inhibitory activity was calculated according to the following formula:

$$\mathrm{Metalloproteinase}\;\mathrm{Inhibitory}\;\mathrm{Activity}\;\left(\%\right)=100\times \left(1-\frac{{\mathrm{Abs}}_{450\mathrm{nm},\;\mathrm{inhibitor}}}{{\mathrm{Abs}}_{450\mathrm{nm},\;\mathrm{negative}\;\mathrm{control}}}\right)$$

The concentration–response curves of the inhibitory activity were plotted in GraphPad Prism 6.0 (GraphPad software, San Diego, CA, USA).

### 4.8. Phospholipase Inhibitory Activity

The effect of varespladib on lipolytic activity of NnNV was determined using a monodisperse synthetic chromogenic substrate 4-nitro-3-octanoyloxybenzoic acid (NOBA) [75]. Briefly, 20 μL of NnNV was premixed with 20 μL of various amounts of varespladib (final concentration, 25 μM–1000 μM) at 37 °C for 60 min. Then, 200 μL of assay buffer (50 mM Tris-HCl, 5 mM CaCl_2_, 100 mM NaCl, pH 8.0) was further added. The reaction was initiated by adding 20 μL of NOBA (1 mg/mL in acetonitrile) into each well. The mixtures were then incubated for another 60 min at 37 °C, and the absorbance at 405 nm was recorded. In negative control, 20 μL of assay buffer was added into 20 μL of NnNV to represent the inhibition of 0%. As a compound control, 20 μL of varespladib was mixed with same volume of assay buffer without adding NnNV, and the absorbance at 405 nm was measured. Assays were performed in triplicate and two replicates were set for compound control at each concentration. The phospholipase inhibitory activity was calculated according to the following formula:

(1) $$\begin{array}{c} \mathrm{Phospholipase}\;\mathrm{Inhibitory}\;\mathrm{Activity}\;\left(\%\right)\\ \;=100\;\times \left(1-\frac{{\mathrm{Abs}}_{405\mathrm{nm},\;\mathrm{inhibitor}}-{\mathrm{Abs}}_{405\mathrm{nm},\;\mathrm{compound}\;\mathrm{control}}}{{\mathrm{Abs}}_{405\mathrm{nm},\;\mathrm{negative}\;\mathrm{control}}}\right) \end{array}$$

The concentration–response curve of the inhibitory activity were plotted in GraphPad Prism 6.0 (GraphPad software, San Diego, CA, USA).

### 4.9. Statistical Analysis

All of the tests are repeated at least three times and results are presented as the mean ± S.E.M. The significance of differences between the means of various experimental groups is analyzed by one-way analysis of variance, followed by a Tukey multiple comparisons test build in GraphPad Prism 6.0 (GraphPad software, San Diego, CA, USA). * *p* < 0.05, ** *p* < 0.01, and *** *p* < 0.001 are considered statistically significant.

## Supplementary Materials

The following materials are available online at www.mdpi.com/2072-6651/9/2/47/s1: Table S1: Identification of the enzymatic constituents in *C. nozakii* nematocysts venom (CnNV) indicated in Figure 4A by liquid chromatography tandem mass spectrometry (LC-MS/MS), Table S2: Identification of the enzymatic constituents in *N. nomurai* nematocyst venom (NnNV) indicated in Figure 4A by liquid chromatography tandem mass spectrometry (LC-MS/MS), Table S3: CfTX-like toxins identified in two scyphozoans *N*. *nomurai* and *C. nozakii* by liquid chromatography tandem mass spectrometry (LC-MS/MS).
