# Supplementary material for: Functional Elucidation of Nemopilema nomurai and Cyanea nozakii Nematocyst Venoms’ Lytic Activity Using Mass Spectrometry and Zymography
Source: Toxins (Basel). 2017 Jan 26;9(2):47. doi: 10.3390/toxins9020047 (PMC5331427; doi:10.3390/toxins9020047)
Supplement: Supplementary file 1 [file toxins-09-00047-s001.pdf]

Supplementary Materials: Functional Elucidation of *Nemopilema nomurai* and *Cyanea nozakii* Nematocyst Venoms' Lytic Activity Using Mass Spectrometry and Zymography

Yang Yue, Huahua Yu, Rongfeng Li, Ronge Xing, Song Liu, Kecheng Li, Xueqin Wang, Xiaolin Chen and Pengcheng Li

1. Supplementary Information

Table S1. Identification of enzymatic constituents in jellyfish *C. nozakii* nematocysts venom (CnNV) indicated in Figure 4A by liquid chromatography tandem mass spectrometry (LC-MS/MS).

Band No.	Venom Family	Protein Name/Source Organism	Mass (Da)	Accession	Protein Score	Sequence Coverage
CnNV-1~66 KDa	Phospholipase D	Phospholipase D(Fragments)/Loxosceles cf. spinulosa	31,968	C0JB40	28	8%
		Phospholipase D SpeSicTox-betaIB4 (Fragment) OS = Sicarius peruensis	31,259	C0JB34	27	13%
		Phospholipase D LbSicTox-betaIA1a OS = Loxosceles boneti	31,921	Q5YD76	19	9%
	Phospholipase A1	Phospholipase A1 OS = Polistes annularis	34,203	Q9U6W0	24	13%
		Probable phospholipase A1 magnifin OS = Vespa magnifica	38,504	P0CH47	14	5%
		Phospholipase A1 OS = Solenopsis invicta	39,190	Q68KK0	13	5%
	Phospholipase A2	Basic phospholipase A2 homolog zhaoermiatoxin O = Protobothrops mangshanensis	14,774	P84776	21	12%
		Basic phospholipase A2 DAV-N6 OS = Deinagkistrodon acutus	16,641	Q1ZY03	18	5%
		hospholipase A2 homolog OS = Echis coloratus	14,495	P0DMT3	17	6%
		Basic phospholipase A2 DsM-b1/DsM-b1′ OS	16,688	A0CG82	16	6%
	Zinc metalloproteinase-disintegrin-like	Zinc metalloproteinase-disintegrin-like OS = Cerberus rynchops	71,078	D8VNS0	20	2%
		Zinc metalloproteinase-disintegrin-like brevilysin H2a	48,693	P0DM89	16	2%
	Other	Thrombin-like enzyme TLBm OS = Bothrops marajoensis	33,903	P0DJE9	19	7%
		Thrombin-like enzyme RP34 (Fragment) OS = Cerastes cerastes	3783	Q9PS28	17	36%
		Hyaluronidase A OS = Vespula vulgaris	39,137	P49370	17	9%

CnNV-2~55 kDa	Phospholipase D	Phospholipase D SpeSicTox-betaIB2a (Fragment) OS = Sicarius peruensis	31,305	C0JB37	40	16%
	Zinc Metalloproteinase-disintegrin-like	Zinc metalloproteinase-disintegrin-like VAP2A = Crotalus atrox	70,157	A4PBQ9	45	7%
		Zinc metalloproteinase-disintegrin-like kaouthiagin-like OS	68,187	D3TTC1	18	2%
		Zinc metalloproteinase-disintegrin-like OS = Cerberus rynchops	71,078	D8VNS0	16	1%
		Snake venom metalloproteinase kistomin OS = Calloselasma rhodostoma	48,157	P0CB14	15	3%
	Phospholipase A1	Phospholipase A1 OS	34,203	Q9U6W0	20	8%
	Phospholipase A2	Phospholipase A2 homolog OS = Echis coloratus	14,495	P0DMTS	20	6%
		Basic phospholipase A2 Bs-N6 OS	16,325	Q6EER4	17	15%
		Basic phospholipase A2 nigroxin B OS = Micrurus nigrocinctus	14,158	P81167	16	16%
	Other	Snake venom serine protease HS114 OS	28,509	Q5W959	17	10%
		Hyaluronidase OS = Echis ocellatus	53,137	A3QVN2	21	7%
		Hyaluronidase conohyal-ad1 (Fragment) OS	39,842	I0CME8	16	4%
		Thrombin-like enzyme TLBm OS = Bothrops marajoensis	33,903	P0DJE9	20	6%
		Thrombin-like enzyme cerastotin	11,329	P81038	13	24%
CnNV-3~45 kDa	Serine protease	Venom serine carboxypeptidase OS = Apis mellifera	53,783	C9WMM5	24	11%
		Snake venom serine protease 3 OS = Trimeresurus gramineus	28,700	O13063	22	4%
	Phospholipase D	Phospholipase D LamSicTox-alphaIC1 (Fragment) OS = Loxosceles amazonica	31,020	C0JAZ9	22	2%
		Phospholipase D LiSicTox-betaIA1i OS = Loxosceles intermedia	34,520	Q2XQ09	20	15%
		Phospholipase D SpaSicTox-betaIIA1 (Fragment) OS = Sicarius patagonicus	31,847	C0JB68	16	8%
		Phospholipase D LspiSicTox-betaIE4i (Fragment) OS = Loxosceles spinulosa	31,591	C0JB46	14	17%
	Zincmetalloproteinase-disintegrin-like	Zinc metalloproteinase-disintegrin-like EoVMP2 OS	71,605	Q2UXQ5	20	1%
		Zinc metalloproteinase-disintegrin-like = Cerberus rynchops	71,078	D8VNS0	17	2%
		Zinc metalloproteinase-disintegrin-like BfMP (Fragment) OS = Bungarus fasciatus	70,381	A8QL48	16	6%

	Phospholipase A1/A2	Phospholipase A1 OS = Polistes annularis PE	34,203	Q9U6W0	19	20%
		Phospholipase A2 Scol/Pla OS = Scolopendra viridis PE	17,461	C1JAR9	20	11%
		Acidic phospholipase A2 BA2 OS = Gloydius halys	14,590	O42190	16	5%
		Phospholipase A2 (Fragment) OS = Bothrops jararaca PE	1752	Q9PRZ0	14	73%
	Peptidase-like enzymes	Venom dipeptidyl peptidase	89,267	B1A4F7	16	2%
	Other	L-amino-acid oxidase (Fragment) OS = Naja atra	51,805	A8QL58	15	2%
		Thrombin-like enzyme cerastotin (Fragments) OS = Cerastes cerastes PE	11,329	P81038	13	11%

Table S2. Identification of the enzymatic constituents in jellyfish *N. nomurai* nematocysts venom (NnNV) indicated in Figure 4A by liquid chromatography tandem mass spectrometry (LC-MS/MS).

Band No.	Venom Family	Protein Name/Source Organism	Mass (Da)	Accession	Protein Score	Sequence Coverage
NnNV-1 > 110 kDa	**Metalloprotease**	Astacin-like metalloprotease toxin 5 (Fragment) OS = Loxosceles gaucho	21,858	P0DM62	29	10%
	**Phospholipase A1/A2**	Phospholipase A1 3 (Fragment) OS = Polistes dominula	35,738	Q6Q250	28	6%
		Acidic phospholipase A2 PA4 OS = Heloderma suspectum	16,200	P80003	18	5%
		Phospholipase A2 homolog OS = Echis coloratus PE	14,495	PODMT3	17	6%
		Phospholipase A2 OS = Mesobuthus tamulus	19,188	Q6T178	17	5%
	**Hyaluronidase**	Inactive hyaluronidase B OS = Vespula vulgaris	40,275	Q5D7H4	17	7%
	***L*-amino acid oxidase**	*L*-amino acid oxidase OS = Cerastes cerastes	58,805	X2JCV5	14	6%
		L-amino-acid oxidase OS = Calloselasma rhodostoma PE	58,583	P81382	13	10%
NnNV-2~66 kDa	**Metalloprotease or protease**	Venom protease OS = Bombus pensylvanicus	27,633	Q7M4I3	25	9%
		Astacin-like metalloprotease toxin 1 OS = Loxosceles intermedia	30,650	A0FKN6	21	10%
		Snake venom metalloproteinase H5 (Fragment) OS = Deinagkistro don acutus	46,518	Q9IAY2	14	5%
		Zinc metalloproteinase-disintegrin-like EoVMP2 OS = Echis ocellatus GN	71,605	Q2UXQ5	14	4%
	**Phospholipase A2/A1**	Phospholipase A2 1 (Fragment) OS = Micrurus tener microgalbineus	1397	P25072	37	100%
		Phospholipase A2 OS = Mesobuthus tamulus	19,188	Q6T178	19	5%
		Basic phospholipase A2 PA-13 OS = Pseudechis australis	14,002	P04057	14	14%

		Basic phospholipase A2 bothropstoxin-2 OS = Bothrops jararacussu	16,553	P45881	15	5%
		Phospholipase A1 1 OS = Polistes dominula	38,390	Q6Q252	19	6%
	**Serine proteinase**	Snake venom serine proteinase 4a OS = Crotalus adamanteus	29,589	J3SDX0	17	10%
		Snake venom serine proteinase 9 OS = Crotalus adamanteus	30,195	J3RYA3	16	3%
	**L-amino-acid oxidase**	*L*-amino-acid oxidase OS = Calloselasma rhodostoma	58,583	P81382	21	12%
		L-amino-acid oxidase OS = Pseudechis australis	59,049	Q4JHE1	15	3%
NnNV-3~46 kDa	**Metalloproteinase**	Zinc metalloproteinase carinactivase-1 catalytic subunit (Fragment) OS = Echis carinatus PE	3748	Q9PRP9	64	37%
		Astacin-like metalloprotease toxin 1 OS = Loxosceles intermedia	30,650	A0FKN6	20	13%
		Snake venom metalloproteinase-disintegrin-like mocarhagin OS = Naja mossambica	70,412	Q10749	18	1%
		Zinc metalloproteinase-disintegrin-like HF3 OS = Bothrops jararaca	69,818	Q98UF9	14	3%
		Snake venom metalloproteinase HT-1 (Fragment) OS = Crotalus ruber ruber	25,182	Q9PSN7	13	5%
	**Phospholipase A2/A1**	Phospholipase A2 1 (Fragment) OS = Micrurus tener microgalbineus	1397	P25072	37	100%
		Basic phospholipase A2 homolog Pgo-K49 OS = Cerrophidion godmani	16,303	Q8UVU7	21	10%
		Acidic phospholipase A2 BA1 OS = Gloydius halys PE	14,847	O42189	19	5%
		Phospholipase A2 (Fragment) OS = Bunodosoma caissarum	4378	P86780	18	33%
	**Phospholipase D**	Phospholipase D LspiSicTox-betaIE4i (Fragment) OS = Loxosceles spinulosa	31,591	C0JB46	19	10%
		Phospholipase D SdSicTox-betaIF1 (Fragment) OS = Sicarius cf. damarensis	32,023	C0JB55	15	5%
	**Serine proteinase**	Snake venom serine proteinase 9 OS = Crotalus adamanteus	30,195	J3RYA3	17	8%
	**Hyaluronidase**	Hyaluronidase A OS = Vespula vulgaris	39,137	P49370	17	4%
		Inactive hyaluronidase B OS = Vespula vulgaris	40,275	Q5D7H4	15	12%
	***L*-amino-acid oxidase**	*L*-amino-acid oxidase OS = Oxyuranus scutellatus scutellatus	59,374	Q4JHE3	22	1%
		L-amino-acid oxidase OS = Vipera ammodytes ammodytes	55,056	P0DI84	17	4%
	Other	Thrombin-like enzyme ancrod-2 OS = Calloselasma rhodostom	29,867	P47797	16	7%

NnNV-4~35 kDa	**Metalloproteinase**	Zinc metalloproteinase carinactivase-1 catalytic subunit (Fragment) OS = Echis carinatus	3748	Q9PRP9	32	37%
		Astacin-like metalloprotease toxin 1 OS = Loxosceles intermedia	30,650	A0FKN6	19	10%
		Zinc metalloproteinase/disintegrin (Fragment) OS = Trimeresurus gramineus	50,111	P0C6E8	15	4%
		Zinc metalloproteinase-disintegrin-like brevilysin H2a	48,693	P0DM89	15	2%
		Zinc metalloproteinase-disintegrin-like Eoc1 OS = Echis ocellatus	70,873	Q2UXR0	13	1%
	**Phospholipase A1**	Phospholipase A1 OS = Solenopsis invicta	39,190	Q68KK0	19	4%
	**Hyaluronidase**	Inactive hyaluronidase B OS = Vespula vulgaris	40,275	Q5D7H4	1	20%
NnNV-5~27 kDa	**Metalloproteinase**	Zinc metalloproteinase carinactivase-1 catalytic subunit (Fragment) OS = Echis carinatus	3748	Q9PRP9	24	37%
		Venom metalloproteinase 2 OS = Eulophus pennicornis	46,696	B5AJT3	21	4%
		Zinc metalloproteinase-disintegrin-like stejnihagin-A OS = Trimeresurus stejnegeri	69,984	Q3HTN1	20	3%
		Zinc metalloproteinase-disintegrin-like berythractivase OS = Bothrops erythromelas	70,767	Q8UVG0	17	1%
		Zinc metalloproteinase-disintegrin-like OS = Cerberus rynchops PE	71,078	D8VNS0	17	2%
		Zinc metalloproteinase-disintegrin-like brevilysin H2a OS = Gloydius brevicaudus	48,693	P0DM89	15	12%
	**Phospholipase D**	Phospholipase D LamSicTox-alphaIC1 (Fragment) OS = Loxosceles amazonica	31,020	C0JAZ9	32	2%
		Phospholipase D SpaSicTox-betaIIA1 (Fragment) OS = Sicarius patagonicus	31,847	C0JB68	18	2%
		Phospholipase D LiSicTox-betaIA1i OS = Loxosceles intermedia	34,520	Q2XQ09	14	7%
	**Phospholipase A2**	Phospholipase A2 1 (Fragment) OS = Micrurus tener microgalbineus	1397	P25072	36	50%
		Basic phospholipase A2 homolog zhaoermiatoxin OS = Protobothrops mangshanensis	14,774	P84776	22	26%
		Basic phospholipase A2 homolog Cax-K49 OS = Crotalus atrox	16,385	Q8UVZ7	19	10%
	***L*-amino-acid oxidase**	*L*-amino-acid oxidase OS = Notechis scutatus scutatus	59,363	Q4JHE2	15	8%

NnNV-6~18 kDa	**Phospholipase A2**	Phospholipase A2 1 (Fragment) OS = Micrurus tener microgalbineus	1397	P25072	37	50%
		Basic phospholipase A2 homolog OS = Protobothrops mucrosquamatus	14,598	P22640	14	12%
	**Phospholipase D**	Phospholipase D SpeSicTox-betaIB1a (Fragment) OS = Sicarius peruensis	31,246	C0JB35	30	9%
		Phospholipase D LiSicTox-alphaIA2ai OS = Loxosceles intermedia	34,402	P0CE83	24	17%
		Phospholipase D StSicTox-betaIC1 (Fragment) OS = Sicarius terrosus	31,271	C0JB39	19	12%
		Phospholipase D SpaSicTox-betaIF1 (Fragment) OS = Sicarius patagonicus	30,636	C0JB52	19	2%
		Sphingomyelin phosphodiesterase D OS = Ixodes scapularis GN	41,826	Q202J4	13	5%
	**Hyaluronidase**	Hyaluronidase OS = Loxosceles intermedia	46,852	R4J7Z9	17	1%
		Hyaluronidase A OS = Vespula vulgaris	39,137	P49370	16	5%
	Other	Zinc metalloproteinase-disintegrin-like brevilysin H2a OS = Gloydius brevicaudus	48,693	P0DM89	14	4%
		L-amino-acid oxidase (Fragment) OS = Naja atra	51,805	A8QL58	13	5%
		Trehalase OS = Pimpla hypochondriaca GN	66,587	Q8MMG9	13	5%

Notes: In Tables S1 and S2, a discrepancy between the observed molecular weights and predicted molecular weights was noticed, which was partially attributed to absence of enough similar sequences with jellyfish-derived enzymes in animal toxin annotation project.

Table S3. CfTX-like toxins identified from two scyphozoans *N. nomurai* and *C. nozakii* by liquid chromatography tandem mass spectrometry (LC-MS/MS).

Toxins	Jellyfish Species	Gel Band No. [a]	Observed Mass [b] (kDa)	MS^2			
				Matches [c] (*n*)	Coverage (%)	Score	Access Number
CfTX-1	*N. nomurai*	NnNV-3	~46	4(1)	8	18	A7L0035
CfTX-2	*N. nomurai*	NnNV-4	~35	2(1)	3	14	A7L0036
	C. nozakii	CnNV-2	~50	7(1)	11	15	A7L0036
		CnNV-3	~46	8(2)	9	25	

[a] Gel band No. is indicated in Figure 4A; [b] Observed mass was evaluated from 12% SDS-PAGE gel under non-reducing conditions; [c] match peptides, number in bracket indicated the number of statistically significant unique peptides.
